# A machine learning algorithm to predict treatment effectiveness for Kawasaki disease in China: a retrospective model development and validation study

**DOI:** 10.3389/fimmu.2025.1629600

**Published:** 2025-11-26

**Authors:** Xuemei Li, Zihan Zhou, Jingyi Fan, Lin Zhao, Ruidi Xu, Dong Li, Xu Ma, Lu Sun, Yujian Wu, Zhouping Wang, Ce Wang

**Affiliations:** 1 Department of Pediatrics, Shengjing Hospital of China Medical University, Shenyang, Liaoning, China; 2 Department of Pediatric Cardiology, Guangzhou Women and Children’s Medical Center, Guangzhou Medical University, Guangzhou, Guangdong, China

**Keywords:** Kawasaki disease, IVIG resistance, machine learning, SHAP algorithm, intravenous immunoglobulin

## Abstract

**Background:**

Kawasaki disease (KD) is the primary cause of acquired heart disease in children. Intravenous immunoglobulin (IVIG) is the first-line therapy for KD; however, IVIG resistance can occur. Reliable treatment efficacy prediction tools for Chinese patients are lacking, which this study aimed to address.

**Methods:**

This retrospective cohort study enrolled patients diagnosed with KD admitted to Shengjing Hospital of China Medical University and collected data on 36 demographic, clinical, and laboratory parameters. Least Absolute Shrinkage and Selection Operator (LASSO) regression was used to identify key predictive variables. The dataset was divided into training (70%) and validation (30%) sets. Ten models were trained through 10-fold cross-validation, and the training set data were balanced using the ROSE method for oversampling. The performance of each model was evaluated using the area under the receiver operating characteristic curve (AUC), sensitivity, specificity, and accuracy. Patients with KD admitted to Guangzhou Women and Children’s Medical Centre, Guangzhou Medical University, between January 2023 and December 2024 were enrolled as an external validation cohort.

**Results:**

The CatBoost machine learning algorithm achieved the best comprehensive results (AUC: 0·960; sensitivity: 0·883; specificity: 0·889, and accuracy: 0·887). The internal validation results with CatBoost were AUC: 0·862; 95% confidence interval [CI]: 0·6453–0·7651; sensitivity: 0·716; specificity: 0·877; and accuracy: 0·861. The external validation results were AUC: 0·834; 95% CI: 0·783–0·884; sensitivity: 0·817; specificity: 0·838, and accuracy: 0·835.

**Conclusions:**

We present a machine learning model that can predict the risk of IVIG non-responsiveness in patients with KD in China. This model may help doctors develop personalized treatment strategies, thus improving the prognosis of KD.

## 1 Background

Kawasaki disease (KD) is an acute vasculitis that primarily affects children under five years old (1). KD primarily manifests as non-exudative bilateral conjunctival hyperemia, oral mucosal changes, non-purulent cervical lymphadenopathy, polymorphic rash, and acute-phase erythema or edema of the hands and feet, or subacute-phase periungual desquamation. Coronary artery lesions (CALs) are the most frequent complication of KD and the leading cause of acquired heart disease in children (2, 3). High-dose intravenous immunoglobulin (IVIG; 2 g/kg) significantly reduces the risk of KD and CALs (4, 5). However, 10–20% of patients do not respond to IVIG, substantially increasing the risk of developing CALs (6, 7). Moreover, more than one IVIG treatment places economic strain on the family. Thus, a tool to accurately predict IVIG non-responders could help develop early intervention strategies, implement more effective alternative treatments for high-risk patients, improve prognosis, and reduce economic pressures. Some scoring models for predicting IVIG resistance exist (8–10), but their practical application is limited, particularly in Northeast China. To date, prediction models for Chinese patients have been developed using regional populations, not national populations, and have not been externally validated.

Machine learning algorithms are advantageous in the medical field given their efficient data processing capabilities, excellent pattern recognition and prediction functions, and ability to handle complex non-linear relationships (11). More specifically, the pattern recognition and prediction performance of machine learning algorithms are remarkable, as they can identify hidden patterns in complex datasets and predict the likelihood of disease development and treatment effects, which has valuable implications for early disease prevention and the development of personalized treatment plans. Medical data are often multidimensional and involve complex non-linear relationships, and machine learning algorithms are particularly well-suited for handling this type of data because they can uncover complex interactions and effects among variables. However, this is difficult to achieve using traditional statistical methods.

Few machine learning applications have been developed for KD, and none are available on a national scale (12). Therefore, this study used advanced machine learning models to predict cases of IVIG resistance in KD.

## 2 Methods

### 2.1 Study population

Patients diagnosed with KD or incomplete KD from January 2012 to December 2024 in the Pediatric Department of Shengjing Hospital of China Medical University were retrospectively identified using the hospital’s electronic medical record system to generate the training and internal validation cohorts. Additionally, patients with KD admitted to Guangzhou Women and Children’s Medical Centre between January 2023 and December 2024 were enrolled as an external validation cohort. The requirement for informed consent was waived because this was a retrospective study.

Patients with KD were identified according to the 2017 American Heart Association guidelines (4). A classic KD diagnosis requires a fever lasting at least five days and at least four of the following clinical signs: non-exudative bilateral bulbar conjunctival injection; oral mucosal changes (such as red lips, strawberry tongue, or diffuse erythema of the oral and pharyngeal mucosa); non-suppurative cervical lymphadenopathy; polymorphous rash; and erythema or edema of the hands and feet during the acute phase or periungual desquamation in the subacute phase. The diagnostic criteria for incomplete KD: fever lasting five or more days is accompanied by only two or three principal clinical criteria. In such cases, the diagnosis is confirmed by supplemental laboratory criteria or the presence of coronary artery abnormalities detected by echocardiography.

To ensure KD was accurately diagnosed—especially in cases with clinical overlap—we implemented a three-step confirmation process: All patients, irrespective of infection status, were required to fulfill the 2017 AHA KD diagnostic criteria. Each case underwent systematic echocardiography to assess coronary artery status. Final diagnosis was confirmed by a pediatric rheumatologist or infectious disease specialist, who actively excluded alternative diagnoses based on overall clinical presentation.

Patients receiving irregular treatment, those receiving IVIG therapy at other institutions before admission, and those not receiving IVIG during hospitalization were excluded. Patients with severe coexisting systemic diseases (e.g. allergic vasculitis, systemic juvenile idiopathic arthritis, or macrophage activation syndrome), severe hematopoietic or immune system impairment, or comorbid malignant neoplasms were also excluded.

### 2.2 Definitions

IVIG-resistant KD (IVIGRKD) was defined as the recrudescence of fever (axillary temperature ≥37.3 °C) at any time ≥36 hours after the completion of the first IVIG infusion, accompanied by at least one of the five primary clinical manifestations of KD.

### 2.3 Data collection

The following data were collected from our hospital’s electronic medical record database:

1. General information: age (in months), sex, and admission date.
2. Clinical information: primary clinical manifestations (rash, Bacillus Calmette-Guérin [i.e. BCG] inoculation site erythema, conjunctival congestion, orofacial changes, enlarged cervical lymph nodes, erythema and edema of the hands and feet during the acute phase, or periungual desquamation in the subacute phase), fever duration, fever duration before the first IVIG dose and the presence of IVIGRKD.
3. Laboratory findings before the initial IVIG treatment: routine blood count (neutrophil percentage, white blood cell count, platelet count, hemoglobin level), erythrocyte sedimentation rate, and C-reactive protein, N-terminal brain natriuretic peptide (NT-proBNP) precursor, serum albumin (ALB), alanine aminotransferase, serum sodium ion, and serum total bilirubin (TBIL) levels. In cases where multiple measurements were available before the initial treatment, the maximum value was selected for neutrophil percentage, platelet count, white blood cell count, erythrocyte sedimentation rate, and C-reactive protein, NT-proBNP precursor, alanine aminotransferase, and serum TBIL levels. The minimum value was selected for serum sodium ion, serum ALB, and hemoglobin levels.
4. Infectious indices: presence of infection, mixed infections, mycoplasma, streptococcus, Epstein-Barr virus, herpes simplex, rotavirus, norovirus, adenovirus, parainfluenza virus, chlamydia, and coxsackievirus. Method of Infection Documentation: Pathogen detection was performed using a standardized protocol: PCR was used for Mycoplasma, Chlamydia, Epstein–Barr virus (EBV), cytomegalovirus (CMV), coxsackievirus (pharyngeal swab), and norovirus (stool). Rapid antigen testing was applied for group A streptococcus (throat) and rotavirus (stool); streptococcal cases also required supportive clinical features or confirmatory culture. All testing was conducted on samples collected within 1 week before or after hospital admission. Temporal Link to KD Onset: We defined a “concurrent infection” as a pathogen detected within the 7-day window surrounding admission (i.e., from 7 days before to 7 days after). This interval was chosen to capture infections closely associated with the acute febrile phase of KD, minimizing the inclusion of incidental or remote positives.

Clinical data for the external validation cohort included winter onset, chlamydial infections, conjunctival congestion, fever duration, IVIGR resistance status, and hemoglobin, NT-proBNP, serum ALB, and TBIL levels.

### 2.4 Statistical analyses

Statistical analyses were performed using R version 4.4.0 (R Core Team, Vienna, Austria). Categorical variables were analyzed using the χ² test; data are presented as frequencies (%). Non-normally distributed continuous variables were analyzed using the Mann–Whitney U test; data are summarized as medians and interquartile ranges. Statistical significance was set at p <0.05. Least Absolute Shrinkage and Selection Operator (LASSO) and multivariate logistic regression analyses were employed to identify the independent risk factors for IVIGRKD. The dataset was divided into training (70%) and testing (30%) sets. The ROSE method was applied for oversampling to address the class imbalance. All machine learning models were trained on the training set, including logistic regression, support vector machine (SVM), extreme gradient boosting (XGBoost), random forest, artificial neural network, gradient boosting machine (GBM), AdaBoost, LightGBM, CatBoost, and SVM with polynomial kernels. Receiver operating characteristic (ROC) curves were generated to assess the predictive performance of each model based on the area under the curve (AUC), sensitivity, specificity, accuracy, and 95% confidence interval (CI).

For the external validation cohort, severe missing values were deleted, and samples with fewer missing values were imputed (mean imputation was used for continuous variables and mode imputation was used for categorical variables).

### 2.5 Shapley Additive Explanations algorithm

The SHAP algorithm is a game theory-based graphical tool for interpreting machine learning model outputs (13). This approach is applied to overcome the ‘black box’ characteristics of machine learning, resulting in consistent model interpretability. Additionally, this method provides a clear framework for assessing the significance of individual features by quantifying their contributions to the model’s predictions. It identifies the influence of each feature on the results and offers a global interpretation that helps explain the model’s behavior as a whole. For instance, an interpretable machine learning approach for predicting long-term cardiovascular disease risk in teenagers was generated using the SHAP algorithm (14), providing a novel way to inform public health policies and clinical decision-making. Additionally, one study (15) provided a comprehensive evaluation of interpretable machine learning methods applied to electrocardiogram-based heart disease classification, which led to the development of a new explanatory framework for the early detection of heart disease. We used the SHAP algorithm to analyze the global and individual sample variables of the best-fit model to quantitatively visualize the relationship between risk factors and outcomes, thus enhancing the model’s credibility.

## 3 Results

### 3.1 Basic information

We retrospectively identified 5,218 patients with KD, of whom 4,704 met the inclusion and exclusion criteria and were enrolled in the study (2,901 boys and 1803 girls; male-to-female ratio: 1·63:1). The median age at disease onset was 25 months (interquartile range, 14–40 months). Patients whose body temperature normalized within 36 h after the first infusion were considered responsive to the IVIG treatment; 4,231 patients (89·9%) were responsive (i.e. IVIG-sensitive), and 473 (10·1%) were not (i.e. IVIGRKD) (**Table 1**).

**Table 1:** Baseline table of clinical indicators for IVIG non-responsive and IVIG sensitive groups.

Variables	Total (n = 4704)	IVIG Sensitive (n = 4231)	IVIG Non-Responsive (n = 473)	P value
Sex, n (%)				0.498
Female	1803 (38)	1629 (39)	174 (37)	
Male	2901 (62)	2602 (61)	299 (63)	
Season, n (%)				0.011
spring	1017 (22)	919 (22)	98 (21)	
summer	1306 (28)	1190 (28)	116 (25)	
autumn	1302 (28)	1180 (28)	122 (26)	
winter	1079 (23)	942 (22)	137 (29)	
Infection, n (%)	2271 (48)	2061 (49)	210 (44)	0.083
Mix infection, n (%)	923 (20)	827 (20)	96 (20)	0.743
MP, n (%)	1423 (30)	1288 (30)	135 (29)	0.423
CP, n (%)	178 (4)	143 (3)	35 (7)	< 0.001
Streptococcus, n (%)	102 (2)	98 (2)	4 (1)	0.055
EBV, n (%)	226 (5)	199 (5)	27 (6)	0.392
HSV, n (%)	295 (6)	257 (6)	38 (8)	0.117
RV, n (%)	147 (3)	133 (3)	14 (3)	0.938
NV, n (%)	86 (2)	69 (2)	17 (4)	0.004
ADV, n (%)	100 (2)	94 (2)	6 (1)	0.232
Influenza virus, n(%)	578 (12)	524 (12)	54 (11)	0.593
CV, n (%)	146 (3)	126 (3)	20 (4)	0.178
Rash	3532 (75)	3176 (75)	356 (75)	0.969
BCG	739 (16)	660 (16)	79 (17)	0.577
Lips and oral	4030 (86)	3637 (86)	393 (83)	0.105
Hand and foot	2437 (52)	2192 (52)	245 (52)	1
Cer	4176 (89)	3769 (89)	407 (86)	0.057
Con	4143 (88)	3743 (88)	400 (85)	0.016
Per	1834 (39)	1599 (38)	235 (50)	< 0.001
Age	25 (14, 40)	26 (14, 41)	24 (13, 36)	0.047
Initial IVIG days	6 (5, 8)	6 (5, 8)	6 (5, 8)	0.003
Days of fever	7 (6, 8)	7 (6, 8)	9 (7, 11)	< 0.001
WBC (*10^9/L)	13.1 (10.1, 16.98)	13.1 (10.02, 16.8)	14 (10.44, 17.8)	0.001
NE(%)	64 (51.6, 75.78)	63.2 (51.2, 74.4)	73.4 (58.5, 83.1)	< 0.001
HB(g/L)	110 (103, 117)	110 (104, 117)	104 (95, 113)	< 0.001
PLT(*10^9/L)	362 (280, 467)	363 (284, 462)	352 (256, 560)	0.437
ESR (mm/h)	60 (44, 79)	60 (44, 79)	62 (45, 81)	0.15
CRP (mg/L)	55.7 (24.1, 94.81)	52.8 (23.1, 90.3)	87.5 (38.2, 130)	< 0.001
BNP (pg/mL)	371 (146.28, 1044.25)	338.3 (138.85, 908.3)	1029 (304.1, 3264)	< 0.001
ALB(g/L)	34.2 (31.3, 37.5)	34.6 (31.8, 37.7)	30.5 (27.2, 34.3)	< 0.001
ALT (U/L)	20 (12, 51)	20 (12, 48)	31 (14, 78)	< 0.001
TBIL(umol/L)	4.8 (3.3, 7.3)	4.7 (3.3, 7)	6.3 (3.8, 12.5)	< 0.001
Na+(mmol/L)	136 (134, 138)	136 (134, 138)	135 (133, 137)	< 0.001

ADV, Adenovirus; ALB, serum albumin; ALT, alanine aminotransferase; BCG, BCG site erythema; BNP, N-terminal brain natriuretic peptide precursor; Cer, enlarged cervical lymph nodes; Con, conjunctival congestion; CRP,C-reactive protein; ESR, erythrocyte sedimentation rate; CT, Chlamydia; CV, Coxsackievirus; ESR, erythrocyte sedimentation rate; Hand and foot, erythema and edema of the hands and feet during the acute phase; HB, hemoglobin; HPIV: Parainfluenza Virus; HSV, Herpes simplex; Infection, Presence of infection; Initial IVIG days, time span of fever before IVIG administration; Lips and oral, orofacial changes; Mix infection, Presence of mixed infections; MP, Mycoplasma; Na+:serum sodium ion; NE%,percent neutrophils; NV, Norovirus; Per, periungual desquamation in the subacute phase; PLT, platelet count; HB, hemoglobin; RV, Rotavirus; TBIL, serum total bilirubin level; WBC, white blood cell count.

### 3.2 Feature selection

**Figure 1:**
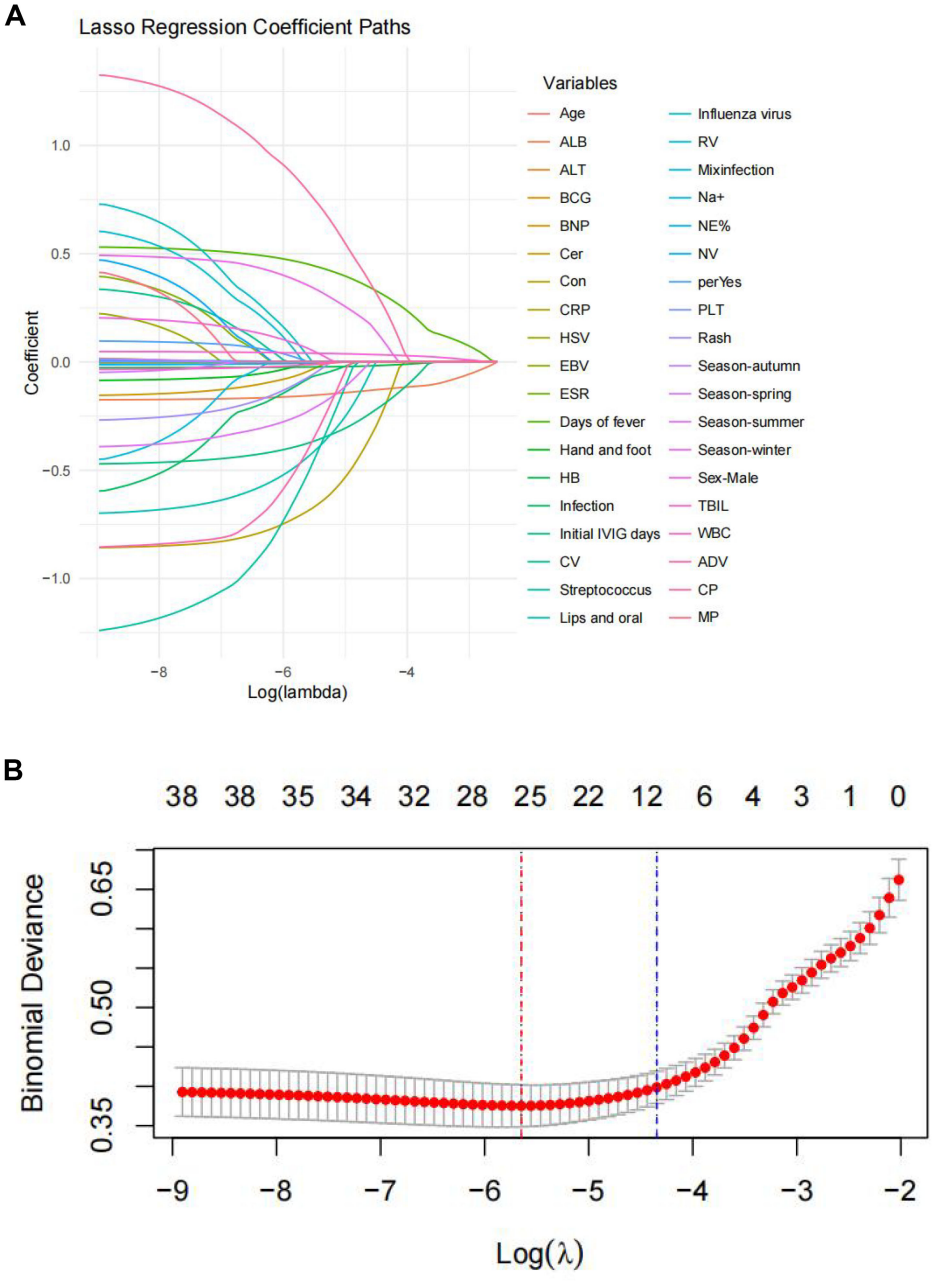
LASSO regression analysis for feature selection in Kawasaki disease. **(A)** shows the LASSO coefficient paths for 36 features; **(B)** the selected λlse that minimizes the mean squared error, identifying 10 main predictive features for the model.

LASSO regression employs L1 regularization (λ penalty term) to shrink the coefficients of less influential variables to zero. This process selects the features with the strongest predictive power for the target variable, simplifying the model and enhancing its generalization capability. We chose the core variables corresponding to lambda.lse, that is, the penalty parameter that minimizes the model’s mean square error (or binomial deviation) during cross-validation; the variables screened by this parameter were used for subsequent model construction. LASSO analysis produced coefficient profiles for 36 features (**Figure 1A**), demonstrating the dynamic process of the LASSO analysis for screening variables. These 38 variables from the training set were included in the LASSO regression analysis, utilizing ten-fold cross-validation for model selection (**Figure 1B**). A λ value (contraction parameter) of 0·009174 was selected based on the cross-validation results, yielding 25 potential predictors, including age, sex, season, the main clinical manifestations of KD (e.g. non-suppurative conjunctivitis, cervical lymphadenopathy, polymorphous rash, acute-phase induration of hands and feet or subacute-phase periungual desquamation), fever duration, timing of the initial IVIG administration, the IVIG inactivation process, the incidence of IVIGRKD, laboratory parameters before the initial IVIG dose (e.g. hematologic indicators [hemoglobin level, platelet counts], erythrocyte sedimentation rate, and NT-proBNP, serum ALB, alanine aminotransferase, and serum TBIL levels), and the presence of concurrent infections, including streptococcus, rotavirus, parainfluenza, chlamydia, and coxsackievirus. Each of these variables has been linked to IVIG resistance.

### 3.3 Univariate and multivariate logistic analyses of KD presenting with prophylactic unresponsiveness

To further screen relevant variables and exclude redundant terms, we performed univariate and multivariate logistic regression analyses based on the variables identified by the LASSO regression analysis. **Table 2** presents the results of the univariate logistic regression analysis, which identified significant associations between several factors and IVIG resistance, including winter onset, chlamydia, fever duration, lips and oral, non-suppurative conjunctivitis, fever duration before the first IVIG dose, and hemoglobin, NT-proBNP, serum ALB, and TBIL levels (all p <0·05). Multivariate analysis confirmed winter, chlamydia infection, conjunctival congestion, fever duration, and hemoglobin, NT-proBNP, ALB, and TBIL levels as independent risk factors, suggesting that these variables may have an independent impact on the outcome variables.

**Table 2:** The result of univariate and multivariate logistic regression and analysis for training set.

Characteristics	Category	Univariate logistic analysis		Multivariate logistic analysis	
		OR (95CI%)	P value	OR (95CI%)	P value
Season	autumn	Ref	Ref	Ref	Ref
	spring	0.96 (0.69-1.35)	0.818	0.92 (0.62-1.36)	0.681
	summer	0.88 (0.64-1.21)	0.425	0.73 (0.50-1.06)	0.097
	winter	1.54 (1.13-2.08)	0.006	1.44 (1.01-2.07)	0.044
CP	Yes	2.64 (1.71-4.07)	<0.001	2.59 (1.52-4.41)	<0.001
Lips and oral	Yes	0.89 (0.65-1.22)	0.475	\	\
Con	Yes	0.70 (0.51-0.97)	0.03	0.46 (0.31-0.68)	<0.001
Days of fever	\	1.23 (1.19-1.27)	<0.001	1.24 (1.19-1.29)	<0.001
HB(g/L)	\	0.95 (0.94-0.96)	<0.001	0.97 (0.96-0.98)	<0.001
BNP(pg/mL)	\	1.00 (1.00-1.00)	<0.001	1.00 (1.00-1.00)	<0.001
ALB (g/L)	\	0.80 (0.78-0.82)	<0.001	0.83 (0.81-0.86)	<0.001
TBIL (umol/L)	\	1.05 (1.04-1.06)	<0.001	1.05 (1.04-1.06)	<0.001
Initial IVIG days	\	0.99 (0.95-1.04)	0.804	\	\

We incorporated the significantly associated variables (p <0·05) from the multivariate logistic analysis into the machine learning models to enhance the prediction and interpretation of the study’s outcome variables.

### 3.4 Model performance

**Figure 2:**
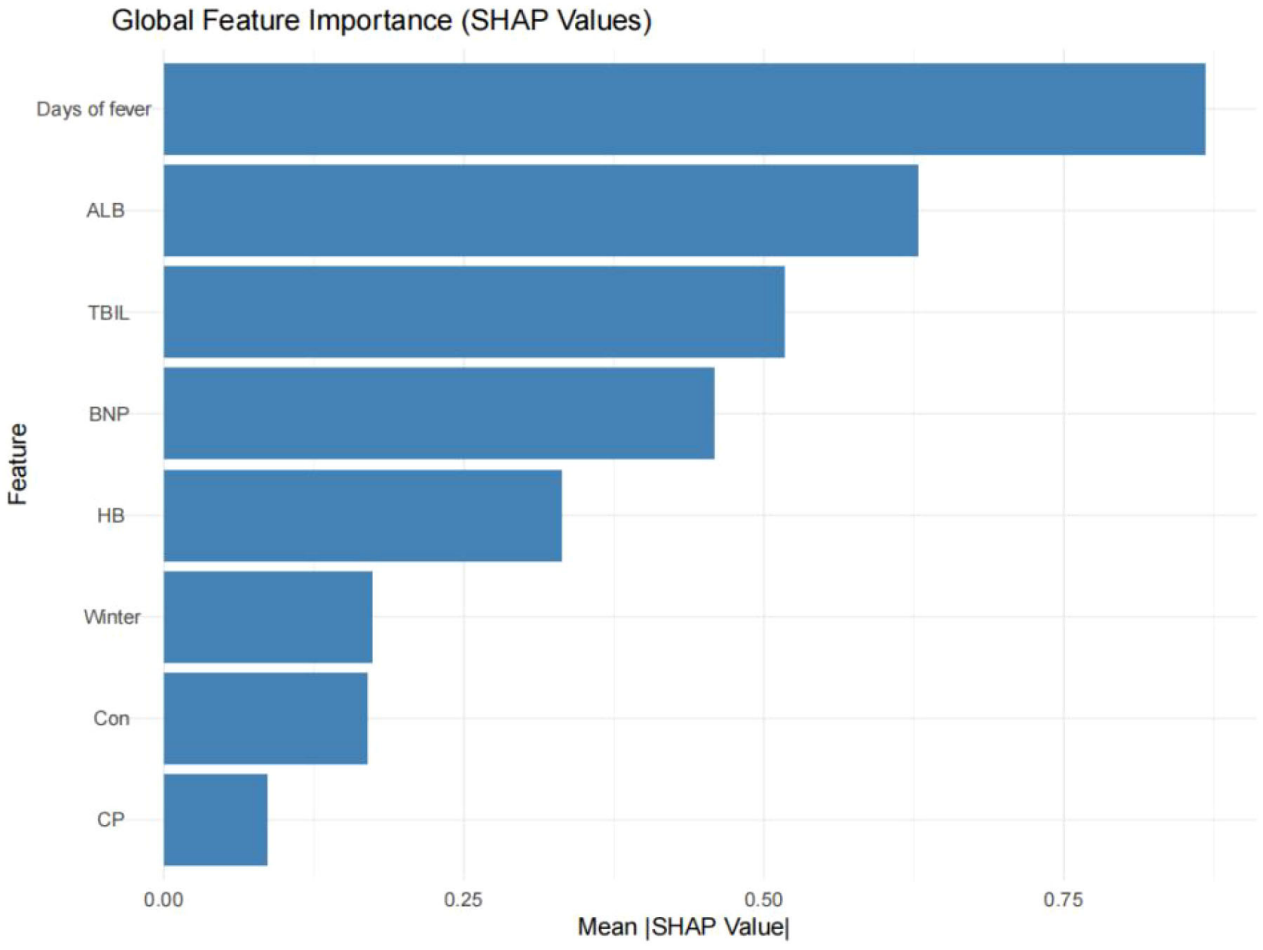
ROC curves for machine learning models predicting IVIG non-response in Kawasaki Disease.

The CatBoost model was the best-fit model based on performance evaluations across the training and validation sets (**Table 3**, **Figure 2**). We trained ten models (logistic regression, SVM, GBM, neural network, random forest, XGBoost, AdaBoost, LightGBM, CatBoost, and polynomial kernel SVM) through 10-fold cross-validation and balanced the training set data using the up-sampling technique. CatBoost achieved the highest AUC (0·960) in the training set, with a highest sensitivity of 0·883 and a highest specificity of 0·889. Furthermore, the overall accuracy of CatBoost was 0·887, indicating strong generalizability. The AUC for CatBoost in the validation set was 0·862 with a 95% CI of 0·827–0·898, indicating its stability with unseen data; the specificity (0·877), sensitivity (0·716), and accuracy (0·861) were also excellent. This balanced and consistent performance made CatBoost the preferred model.

**Table 3:** Performance comparison of ten machine learning (ML) models.

	Model	AUC	95%CI	Sensitivity	Specificity	Accuracy
Training set	Logistic	0.848	0.850-0.869	0.722	0.811	0.767
	SVM	0.892	0.884-0.900	0.775	0.841	0.808
	GBM	0.912	0.905-0.919	0.790	0.838	0.814
	NeuralNetwork	0.861	0.852-0.870	0.737	0.811	0.774
	RandomForest	0.881	0.872-0.889	0.763	0.829	0.796
	XGBoost	0.931	0.925-0.937	0.809	0.869	0.839
	AdaBoost	0.923	0.916-0.929	0.804	0.853	0.829
	LightGBM	0.932	0.927-0.939	0.826	0.844	0.835
	CatBoost	0.960	0.956-0.964	0.883	0.889	0.887
	SVMPoly	0.876	0.867-0.885	0.705	0.856	0.782
Testing set	Logistic	0.831	0.794-0.870	0.730	0.809	0.801
	SVM	0.836	0.798-0.875	0.716	0.823	0.812
	GBM	0.853	0.816-0.889	0.745	0.823	0.815
	NeuralNetwork	0.832	0.795-0.870	0.709	0.805	0.795
	RandomForest	0.832	0.794-0.871	0.723	0.818	0.809
	XGBoost	0.855	0.819-0.892	0.702	0.856	0.840
	AdaBoost	0.847	0.809-0.885	0.738	0.844	0.833
	LightGBM	0.858	0.821-0.894	0.745	0.834	0.825
	CatBoost	0.862	0.827-0.898	0.716	0.877	0.861
	SVMPoly	0.830	0.790-0.870	0.660	0.839	0.821

### 3.5 Interpretation of the CatBoost model based on the SHAP analysis

#### 3.5.1 Global sample feature interpretation

**Figure 3:**
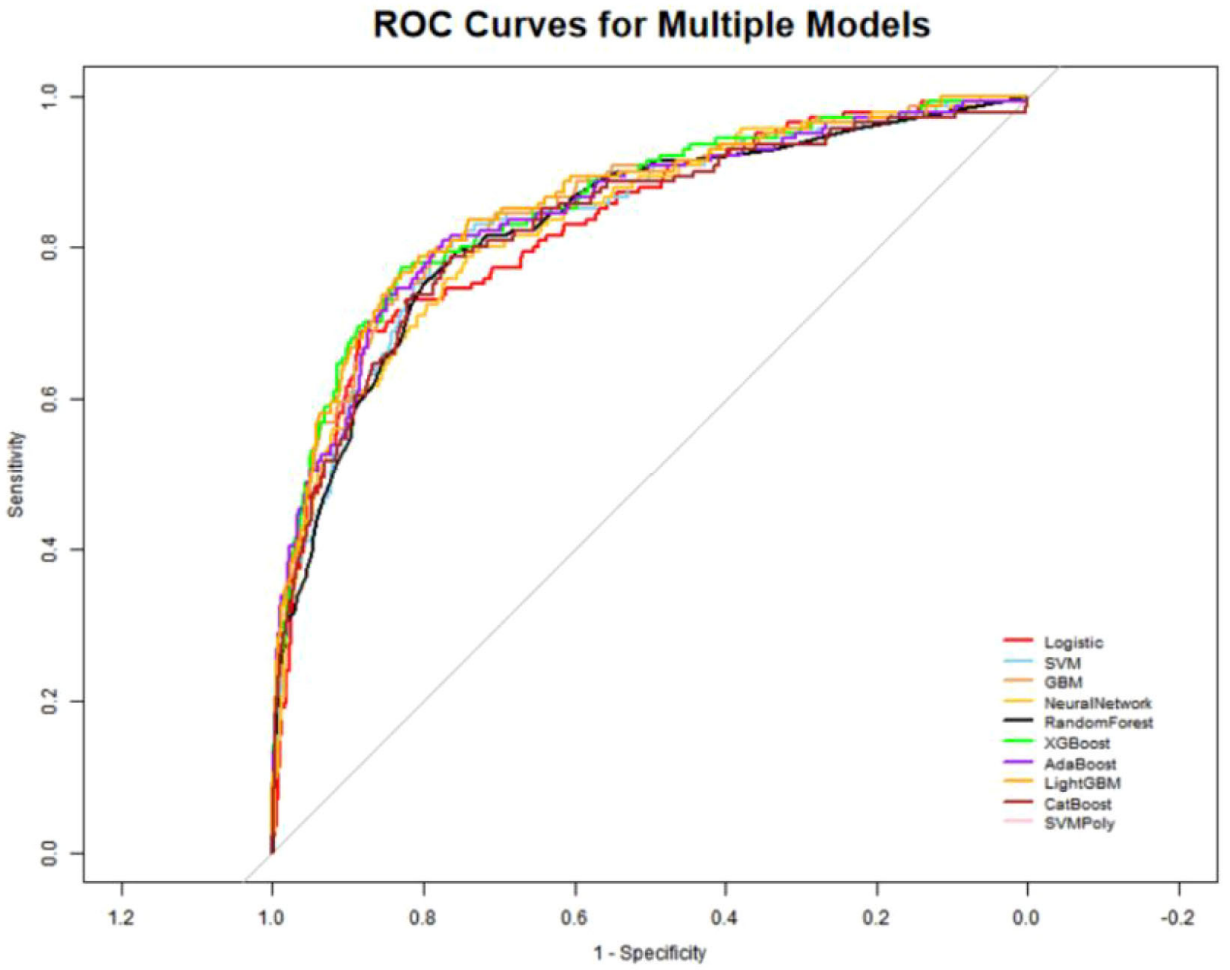
SHAP feature importance analysis of the CatBoost model.

**Figure 4:**
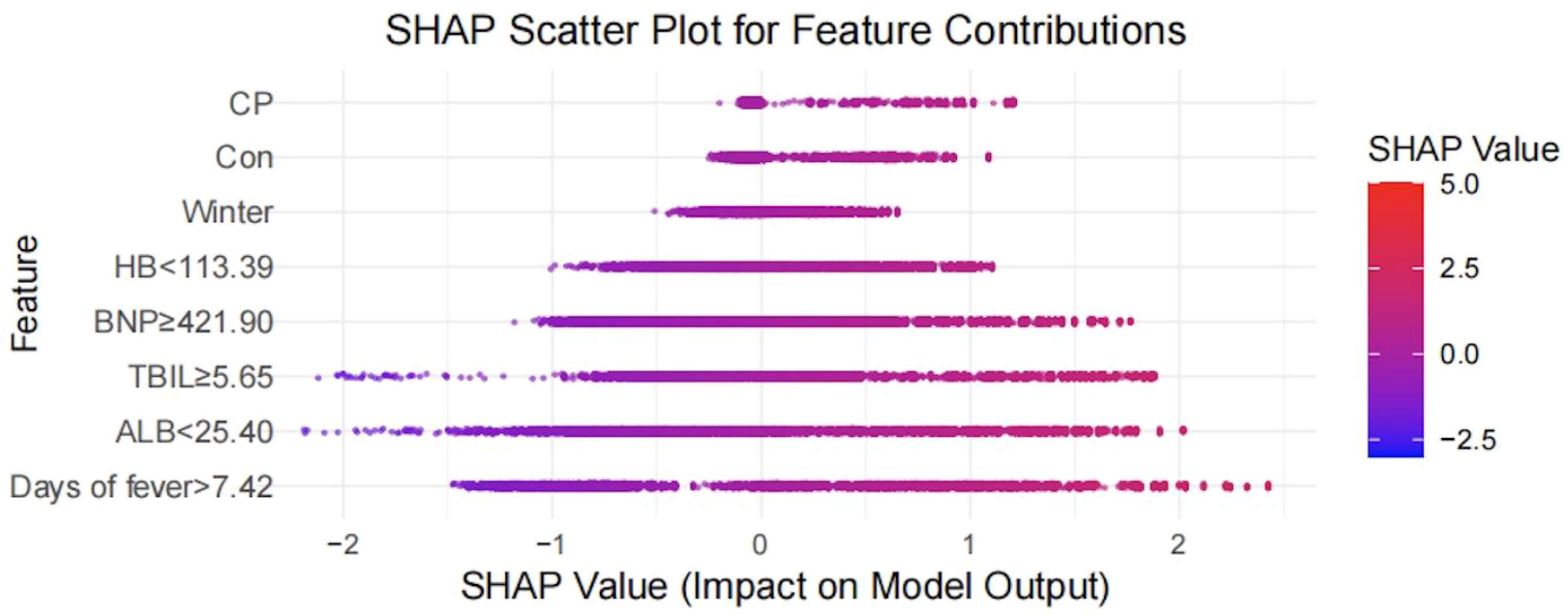
SHAP summary plot illustrating feature effects on IVIG non-response prediction.

**Figure 3** presents the SHAP feature importance ranking for the CatBoost model, illustrating the influence of various features on IVIG non-response predictions in patients with KD. The most influential predictors were fever duration and serum TBIL and ALB levels. Additionally, the SHAP contribution plot (**Figure 4**) revealed how these features affected individual risk predictions; the red dots represent a higher risk, whereas the blue dots represent a lower risk. **Figure 4** displayed the value for each variable at which the predicted probability of IVIG resistance increases most steeply (i.e., the point where the first derivative of the SHAP value curve is maximized). These “maximum-slope” decision points are: Fever Duration: 7.42 days; Albumin: 25.40 g/L; Hemoglobin: 113.39 g/L; Total Bilirubin: 5.65 μmol/L; BNP: 421.90 pg/mL; These values represent quantitative anchors where the risk escalates most rapidly, offering clinical guidance without imposing rigid, binary cut-offs.

#### 3.5.2 Global sample feature interpretation

**Figure 5:**
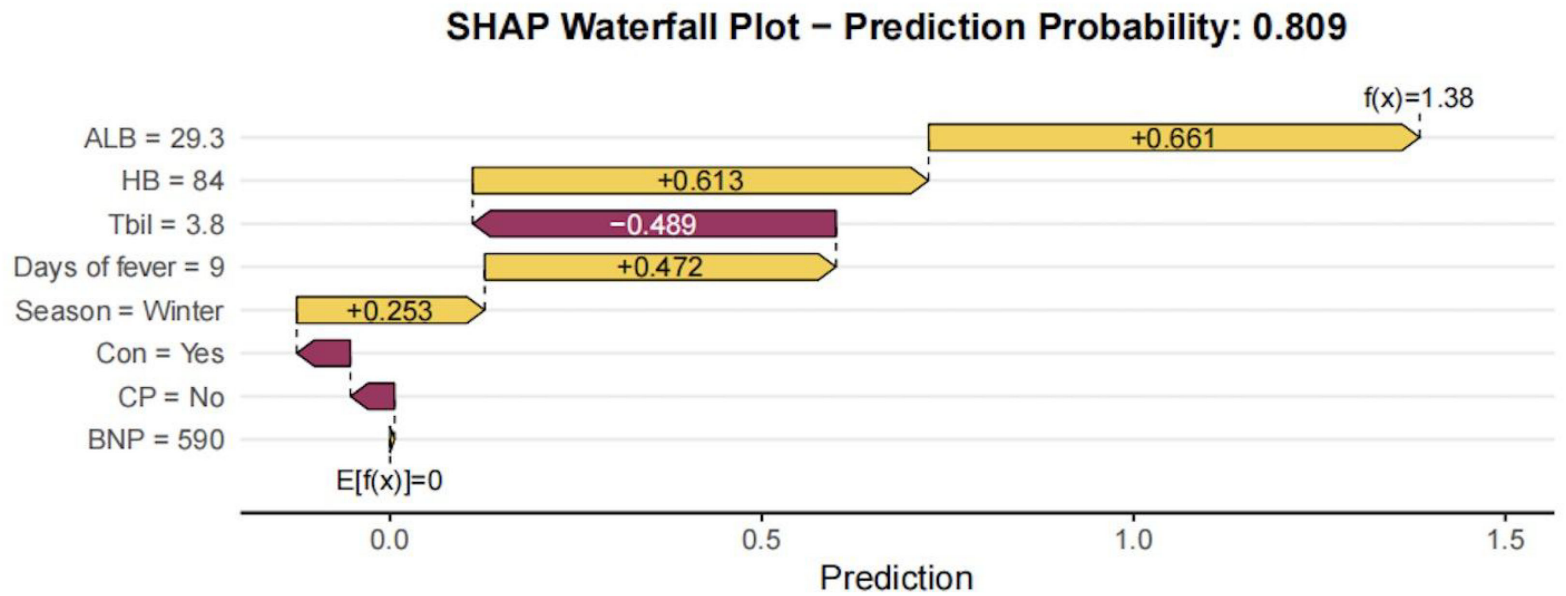
SHAP waterfall plot.

**Figure 5** presents a SHAP waterfall plot. This plot illustrates, for any individual patient, how each feature contributes to shifting the log-odds of IVIG resistance and converts the model output f(x)into a specific probability.

#### 3.5.3 External validation

**Figure 6:**
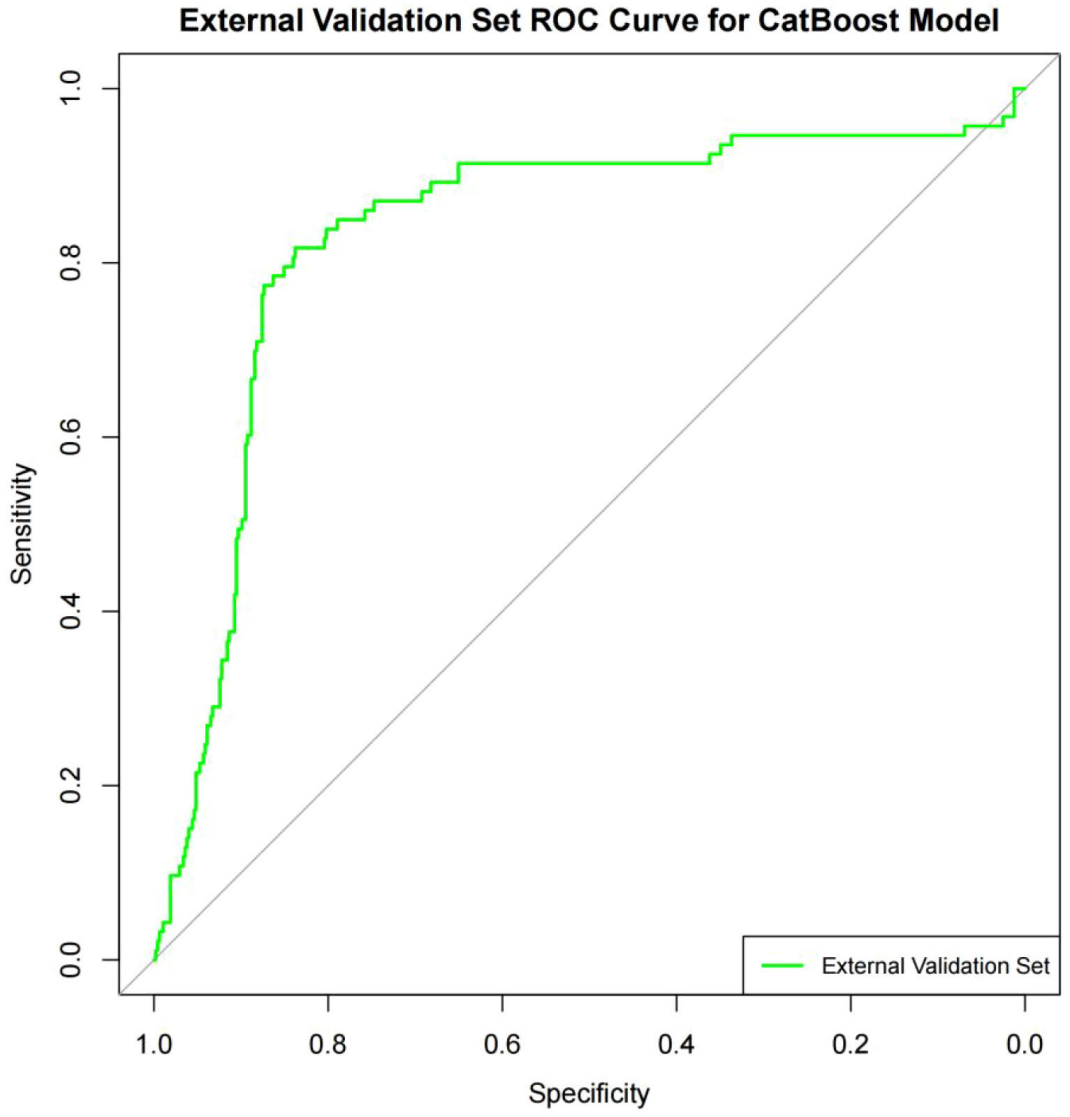
External validation.

The external validation cohort included 568 enrolled patients with KD at Guangzhou Women and Children’s Medical Center, Guangzhou Medical University. These data were used to externally validate the risk prediction model. The sensitivity, specificity, accuracy, and AUC values were 0·817, 0·838, 0·835, and 0·834 (95% CI: 0·783–0·884), respectively (**Figure 6**). These results reflect the model’s long-term continuity and spatial portability and confirm its generalizability and robustness.

## 4 Discussion

Machine-learning algorithms are widely utilized in the diagnosis and prognosis of cardiovascular and cerebrovascular disorders, diabetes, and other conditions (16–18). This study used machine learning algorithms to develop a clinical prediction model for IVIG unresponsiveness in patients with KD for the first time. The CatBoost model, based on a large dataset (n = 4,704) from patients in Northeast China, had the best performance of ten machine learning algorithms. Internal validation of the model using 10-fold cross-validation confirmed its excellent performance and stability, and the external validation results reflect the model’s generalizability and robustness, making the prediction model applicable to the whole country.

This study provides solid support for assessing the risk factors for IVIG unresponsiveness in patients with KD in China. Our evaluation of ten machine learning models resulted in a reliable prediction tool for clinical use that, unlike prior KD prediction models (8–10), was interpretable and could visualize each variable’s impact on the prediction findings. To build the model, key characteristics were screened using LASSO regression to eliminate confounding factors, followed by univariable and multivariable logistic regression analyses. To further understand the fundamental aspects of IVIG resistance, we used the SHAP algorithm to analyze the CatBoost model, which identified eight features with a considerable impact on IVIG resistance: winter onset, chlamydial infections, conjunctival congestion, fever duration, and hemoglobin, NT-proBNP, serum ALB, and TBIL levels. The top three variables influencing the model’s predictions were serum TBIL and ALB levels and fever duration.

CatBoost is a gradient-boosting algorithm for categorical data and high-dimensional features (19). Its intrinsic properties make it one of the best choices for real-world applications dealing with categorical variables efficiently while avoiding time-consuming data preprocessing (20). CatBoost significantly suppresses overfitting and enhances the model’s generalization capabilities by employing a novel stochastic alignment technique, particularly when data are restricted. Compared with other gradient boosting algorithms (for example, XGBoost), CatBoost is more efficient and less demanding for hyperparameter adjustment, trains more quickly, and creates superior models (21). The AUCs for the training and validation sets for our CatBoost model were 0·960 and 0·862, respectively, indicating minimal overfitting and good generalizability. The accuracy, sensitivity, and specificity of the CatBoost model were significantly higher than those of other models, making it more suited for the balanced detection of IVIG-sensitive and IVIG-unresponsive events. Finally, we externally verified our CatBoost risk prediction model using data from 568 prospectively enrolled patients with KD at Guangzhou Women and Children’s Medical Center. The sensitivity, specificity, accuracy, and AUC values were 0·817, 0·838, 0·835, and 0·834 (95% CI: 0·783–0·884), respectively, confirming the model’s generalizability and robustness.

Interestingly, this study discovered for the first time that winter and chlamydial infections significantly enhanced the probability of IVIG resistance, validating the idea that environmental variables affect the IVIG response (22). Winter climate and environmental changes may significantly influence immunological functions, contributing to the development of IVIG resistance in patients with KD. Although winter has previously been identified as the peak season for KD (23), our results imply that an elevated risk of winter onset may be linked to the incidence of winter respiratory infections. Respiratory infection pathogens (e.g. influenza virus and adenovirus) in winter cause an increased inflammatory response, increasing the likelihood of IVIG resistance (24). Chlamydial infections are also more frequent in winter, and the systemic inflammatory response they cause affects the course of KD by stimulating the host’s immune system (25–27). Chlamydial infection induces the release of pro-inflammatory cytokines, such as interleukin (IL)-6 and tumour necrosis factor-alpha (TNF-α), and activates the innate immune response, which subsequently amplifies inflammation, particularly in patients with IVIG-unresponsive KD (28). Chlamydial infection induces a Th1-type immune response, decreasing the anti-inflammatory effects of IVIG (29). Furthermore, immunological changes, such as an active complement system and enhanced macrophage responses during winter, may impair the effectiveness of IVIG (26, 27). Such immune system modifications aggravate the pathogenic potential of KD and reduce the treatment efficacy. Infection-driven hyperinflammatory responses activate multiple immune signaling pathways, including TNF-α and IL-1, which are pivotal in the pathogenesis of IVIG-resistant KD. Infection-induced cytokine release stimulates the nuclear factor kappa-light-chain-enhancer of activated B-cells (NF-κB) pathway, which induces a pro-inflammatory response and causes vascular endothelial damage and coronary artery disease. The NF-κB signaling pathway is related to winter-onset viral infections (30). Therefore, identifying winter onset and chlamydial infection as risk factors for IVIG non-responsiveness provides crucial guidelines for treating children with KD.

We also identified elevated TBIL levels as a risk factor for IVIG resistance in patients with KD. High bilirubin levels may indicate a more severe hepatic inflammatory burden, suggesting disease aggravation (31). Sunaga et al. (32) found that a low TBIL level before IVIG therapy indicated a poor immunological response, which might be related to IVIG resistance. Low bilirubin levels indicate insufficient inflammatory pathway activation, which may result in poor IVIG effectiveness. Children in the Tohoku area may be more prone to cholestasis and hyperbilirubinemia. However, Japanese individuals with Sunaga Y disease exhibit different pathological characteristics. Bilirubin has immunomodulatory properties and acts as an anti-inflammatory agent by blocking T-cell activation and decreasing oxidative stress (33). Low bilirubin levels may be ineffective in reducing excessive inflammation, resulting in IVIG resistance (34). In contrast, elevated bilirubin levels may indicate serious hepatic damage, impairing bile excretion due to increased inflammation and increasing the risk of IVIG therapy failure (35). This biphasic effect may explain the conflicting presentation of TBIL levels and IVIG resistance in several studies.

This study has one key limitation. The development phase data and the external validation data in this study were all collected retrospectively, potentially limiting the ability to control for confounding factors. Future studies should address this issue by including more diverse datasets and using prospective study designs. And this study applied the 2017 AHA diagnostic criteria for consistency. Future prospective validation will include patients diagnosed under the latest standards to further confirm the model’s generalizability.

## 5 Conclusion

This study analyzed the risk factors for IVIG resistance in Chinese patients with KD and constructed a robust predictive CatBoost model with enhanced interpretation via SHAP analysis. Our predictive model performed well and, for the first time, revealed associations between winter-onset KD, Chlamydia infection, and IVIG resistance. We also externally validated the model, demonstrating its generalizability and robustness. This model may help physicians better manage patients with KD, By enabling the early identification of patients at high risk for IVIG resistance, this model facilitates timely treatment intensification. This could involve the initial co-administration of glucocorticoids with IVIG or the prompt initiation of biologic agents upon early signs of treatment failure, rather than awaiting recrudescent fever to administer a second IVIG course. Such a proactive strategy aims to shorten the total duration of inflammation, which is a key driver of coronary artery lesions, thereby potentially reducing the incidence of this serious complication, avoiding the waste of medical resources, and alleviating the economic burden on families.

## Data Availability

The datasets generated and analyzed during the current study are not publicly available due to privacy or ethical restrictions. However, de-identified data are available from the corresponding author upon reasonable request. Requests to access the datasets should be directed to CW, crease.sy@163.com.
